# Obesity at early adulthood increases risk of gastric cancer from the Health Examinees-Gem (HEXA-G) study

**DOI:** 10.1371/journal.pone.0260826

**Published:** 2022-02-04

**Authors:** Hwi-Won Lee, Dan Huang, Woo-Kyoung Shin, Katherine de la Torre, Jae Jeong Yang, Minkyo Song, Aesun Shin, Jong-koo Lee, Daehee Kang

**Affiliations:** 1 Department of Biomedical Sciences, Seoul National University Graduate School, Seoul, Korea; 2 Department of Preventive Medicine, Seoul National University College of Medicine, Seoul, Korea; 3 Integrated Major in Innovative Medical Science, Seoul National University Graduate School, Korea; 4 Department of Family Medicine, Seoul National University Hospital, Seoul, Korea; Children’s Hospitals and Clinics of Minnesota, UNITED STATES

## Abstract

Emerging evidence has indicated a possible link between obesity in early life with subsequent cancer risks, but its association with gastric cancer remains unknown. This study aimed to investigate the association of obesity at ages 18–20 and 35 with the later risk of gastric cancer among the Korean population. Included were 122,724 individuals who participated in the large-scale prospective cohort study, the Health Examinees-Gem (HEXA-G) study, during 2004–2017. Multivariable Cox proportional hazards models were used to estimate hazard ratios (HR) and 95% confidence intervals (CI) for gastric cancer risk associated with body mass index (BMI) at ages 18–20 and 35 years. During a mean follow-up period of 8.6±2.1 years, a total 927 gastric cancer cases (531 men and 396 women) were identified. When compared to normal BMI (18.5–23.0 kg/m^2^), obesity (BMI ≥30 kg/m^2^) at age 35 was significantly associated with increased risk of gastric cancer later in life among total participants (HR 1.94, 95% CI 1.26–2.97, *p* 0.01). When analyzed separately by sex, obesity at 35 years of age was significantly associated with increased risk of gastric cancer among both men (HR 1.79, 95% CI 1.02–3.13, *p* 0.05) and women (HR 2.35, 95% CI 1.21–4.60, *p* 0.02). No significant associations were found for obesity at late adolescence in both men and women. Our findings suggest that obesity in early adulthood may be associated with an increased risk of gastric cancer. The results may aid in understanding the etiology of GC in a population with a divergent trend of gastric cancer.

## Introduction

One of the most common cancers in the world, gastric cancer (GC) accounted for the fifth most frequently diagnosed cancer as well as the third leading cause of cancer death in 2018 [1]. The age-standardized incidence and mortality rates of GC have shown a marked decline globally [2]. However, the absolute number of cases is increasing due to the rapid growth of the aging population. Some countries, including the Republic of Korea, show a divergent trend in the incidence of GC among younger generations [2, 3].

Excess weight is a common risk factor for various health conditions and cancers [4, 5]. The global trend of obesity, or “the obesity epidemic,” is not limited to adults. Increasing rates of obesity at a young age pose a potential burden on our society as a whole and require immediate attention [6]. Development of obesity between early adulthood and midlife is considered a crucial period [7], as studies suggest prolonged obesity over the life-course may be more detrimental to health compared to obesity over a shorter period [8]. Emerging evidence indicates that obesity in early life increases the risk of malignancies, including colorectal, gastroesophageal, pancreatic and ovarian cancer, among others [5, 9–11]. Its association with GC, however, remains largely unknown.

Some cohort studies have suggested a positive association between obesity and GC; yet only a handful have focused on obesity during an earlier time in life [9, 12–16]. Moreover, most of the results have been generated from Western populations. Among the few studies conducted in Asia, a Japanese cohort study reported that high body mass index (BMI) at age 20 years is associated with an increased risk of death from GC [17]. Additionally, some case-control studies have also reported an association between high early adulthood BMI and the risk of GC incidence [18–21], but no single cohort study has yet been reported in Asia. Furthermore, prior studies were mostly focused on adolescent obesity [18], and little is known regarding early adulthood in relation to GC.

Using data from the Health Examinees (HEXA) study, this study aimed to investigate the association between obesity at a younger age (ages 18–20 and 35) and the later risk of GC in a population with a high GC incidence rate.

## Materials and methods

### Study population

**Fig 1:**
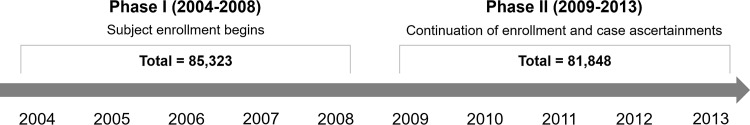
The Health Examinees Study scheme timeline.

This study was conducted among individuals who participated in the baseline survey of the Health Examinees-Gem (HEXA-G) study derived from the Health Examinees study (HEXA), a component of the Korean Genome and Epidemiology study (KoGES). Initiated by the Korean government including National Research Institute of Health, Korea Disease Control and Prevention Agency (KDCA) and the Ministry of Health and Welfare, KoGES is a consortium project of six prospective cohorts categorized into population-based and gene-environment model studies [22]. A part of the population-based cohort category, the HEXA study consists of community dwellers and participants recruited from the national health examinee registry [23]. Participants were enrolled from 38 health examination centers in eight regions across the Republic of Korea between 2004–2013. Participating centers were chosen after strict evaluation of the selection criteria and community representativeness of participating hospitals (Fig 1). After obtaining written informed consent, the study collected data through an interview-based questionnaire, physical examinations as well as laboratory analyses of biological specimens retrieved from study participants. The survey collected information about sociodemographic characteristics, medical history, family history and lifestyle behaviors. The HEXA study continues to follow up on all participants in accordance with a standardized study protocol. It undergoes an annual review by the Ethics Committee of the Korean Health and Genomic Study of the Korea National Institute of Health and the institutional review boards of all participating hospitals (Seoul National University Hospital (IRB number E-2009-117-1159); Korea National Institute of Health (IRB number 2014-08-02-3C-A)). Detailed information on the HEXA study has been described elsewhere [22–24].

In this study, we used the HEXA-G sample defined by the following additional exclusion criteria: 1) Sites that participated in the pilot study only during 2004–2006; 2) Sites that did not meet the HEXA standards for biospecimen quality control; 3) Sites that participated in the study for less than two years [25]. After excluding participants recruited at 21 centers according to the exclusion criteria, 141,899 participants composed of 48,299 men (34.0%) and 93,600 women (66.0%) were included at baseline, and after age restriction (40–69 years), 139,273 participants remained in HEXA-G at baseline.

After merging study results with population cancer registry data, participants without information on the Korea Central Cancer Registry (KCCR; n = 134) and those with prior history of cancer at baseline study (n = 4,679) were excluded.

**Fig 2:**
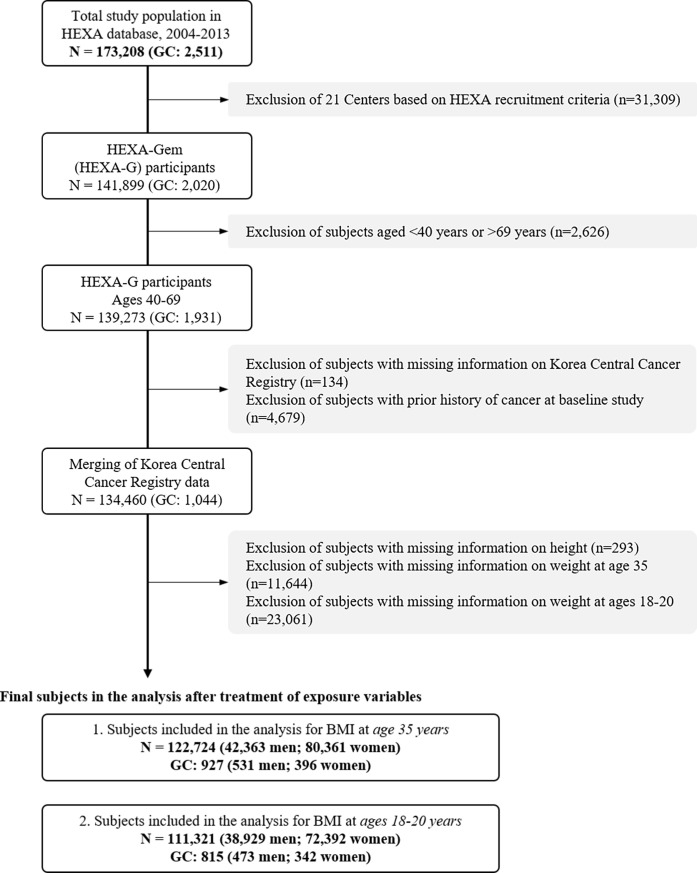
Selection of study participants. HEXA, Health Examinees; GC, gastric cancer; BMI, body mass index.

For the analysis of BMI at age 35, a total of 122,724 participants, including 42,363 men (34.5%) and 80,361 women (65.5%), remained after excluding missing information on height (n = 293) and weight at age 35 (n = 11,644). Additionally, 110,321 participants comprised of 38,929 men (35.3%) and 72,392 women (64.7%) were included in the analysis of the BMI at late adolescence, or ages 18–20, after excluding participants with missing information on weight at 18–20 years (n = 23,061) (Fig 2).

### Ascertainment of gastric cancer

Incident GC cases were identified by linkage to the KCCR data. Provided by the KDCA via collaboration with National Cancer Center Korea, cancer incidence until December 31, 2017 was obtained via resident registration number that is uniquely assigned to each citizen and foreigner residing in the Republic of Korea. The KCCR data is provided to researchers after an extensive review of the study protocol, and all data are fully anonymized before access is granted to each investigator. The primary outcome was defined as the first occurrence of an International Classification of Diseases, 10^th^ Revision (ICD-10) code for GC (C16).

### Obesity at a younger age

At baseline interview, height (cm) and weight (kg) were measured by trained medical staff using standardized KoGES protocol; weight (kilograms) at ages 18 or 20 and 35 years were obtained during baseline interview via self-report. The information obtained through anthropometric measurements and in-person interviews were used to calculate BMI or Quetelet index (weight in kilograms divided by height squared in meters, kg/m^2^). Our study used the Asia-Pacific classification of BMI suggested by World Health Organization (WHO), where a BMI of 18.5–23.0 kg/m^2^ is considered the normal reference range for the Asian population. We further divided the BMI category into 2.5 BMI-unit increments, using the BMI of 22.6–25.0 kg/m^2^ as the reference range recommended according to results from a large pooled analysis for BMI criteria for overweight and obesity in Asians [26]. Moreover, obesity at late adolescence was defined as age range 18–20, and early adulthood was used to describe age 35 years [27].

### Other factors

Using a structured questionnaire, trained interviewers collected data on sociodemographics (age, sex, education and marital status), medical and family history and lifestyle factors (smoking, drinking, physical activity and dietary intake) at enrollment.

### Statistical analyses

To test the differences in the distribution of epidemiologic factors across Asia-Pacific classification BMI category of the participants included in the analysis for BMI at 35 years old, ANOVA and Chi-square tests were used for continuous variables and categorical variables, respectively. To reflect sex differences in epidemiologic risk factors, we performed separate analyses for men and women. We used Cox proportional hazards models to estimate adjusted hazard ratios (HRs) and 95% confidence intervals (CIs) to associate obesity with GC incidence. Covariates for multivariable HRs included sex, education (middle school or less, high school diploma, college degree or higher), smoking status (never/former/current), drinking status (never/former/current), family history of GC, physical exercise (regular exercisers/non-exercisers) and total energy intake (kcal/day, continuous). Tests of linear trend were conducted using median values of each BMI category entered as continuous variables across BMI categories.

The follow-up of the study participants occurred from the time at the completion of the baseline study until the date of GC diagnosis, loss of follow-up, or last date of follow-up (December 31, 2017), whichever came first. All statistical analyses were performed using SAS software version 9.4 (SAS Institute, Cary, NC, USA) and were considered statistically significant with *p* values less than 0.05.

## Results

Among 122,724 participants (42,363 men and 80,361 women) included in the analysis for BMI at age 35 years, 927 GC cases (531 (57.3%) men and 396 women (42.7%) were identified during a mean follow-up period of 8.6±2.1 years through December 31, 2017.

Tables 1 and 2 provide baseline characteristics of men and women, respectively, in the analysis for BMI at age 35. In both sexes, more people (42.1% of men, 62.1% of women) had a BMI range of 18.5–23.0 kg/m^2^. The proportion of men in the lowest (<18.5 kg/m^2^) and highest categories of BMI (≥30.0 kg/m^2^) at age 35 were 1.2% and 1.7%, respectively; among women, there was a relatively greater portion of participants in the lowest BMI category than highest category of BMI at age 35 (5.7% and 1.0%, respectively). Higher proportion of men were married in all five BMI categories, and higher proportions of ever drinkers and men with higher total energy intake were observed among overweight or obese men (BMI >25.0 kg/m^2^). Women with lowest BMI had higher proportions of ever smokers and regular exercisers, while those with highest BMI were found to have lowest educational attainment.

**Table 1:** Baseline characteristics of the HEXA-G study population included in the analysis for GC and BMI at 35 years (Men).

Characteristics	Men (N = 42,363)										
	<18.5 kg/m^2^	(%)	18.5–23.0 kg/m^2^	(%)	23.0–25.0 kg/m^2^	(%)	25.0–29.9 kg/m^2^	(%)	≥30.0 kg/m^2^	(%)	*p* ^a^
Number of participants	514	(1.2)	17,816	(42.1)	13,193	(31.1)	10,123	(23.9)	717	(1.7)	<0.001
Number of participants BMI at age 18–20	466	(1.2)	16,021	(41.9)	11,946	(31.2)	9,146	(23.9)	663	(1.7)	<0.001
Follow-up year (mean ± SD)	8.7	(2.1)	8.7	(2.0)	8.6	(2.0)	8.6	(2.0)	8.5	(2.0)	0.002
Age (mean ± SD)	52.8	(8.7)	53.4	(8.2)	53.5	(8.3)	52.9	(8.5)	51.2	(8.6)	<0.001
Family history of gastric cancer	42	(8.2)	1,534	(8.6)	1,080	(8.2)	768	(7.6)	55	(7.7)	0.151
Education											0.321
≤Middle school	110	(21.4)	3,736	(21.0)	2,742	(20.8)	2,009	(19.9)	135	(18.8)	
High school diploma	205	(39.9)	7,360	(41.3)	5,353	(40.6)	4,224	(41.7)	316	(44.1)	
≥College degree	193	(37.6)	6,542	(36.7)	4,985	(37.8)	3,790	(37.4)	258	(36.0)	
Marital status, Married/cohabitation	466	(90.7)	16,708	(93.8)	12,486	(94.6)	9,535	(94.2)	662	(92.3)	<0.001
Smoking status, Ever	354	(68.9)	13,021	(73.1)	9,536	(72.3)	7,372	(72.8)	528	(73.6)	0.386
Alcohol drinking status, Ever	371	(72.2)	14,040	(78.8)	10,711	(81.2)	8,316	(82.2)	584	(81.5)	<0.001
Physical activity, Regular exercisers	302	(58.8)	8,139	(45.7)	5,408	(41.0)	3,923	(38.8)	280	(39.1)	<0.001
Total energy intake (kcal, mean ± SD)	1774.6	(570.5)	1838.9	(541.6)	1870.6	(563.8)	1906.8	(588.9)	1998.2	(696.8)	<0.001

HEXA-G, Health Examinees Study-Gem; GC, gastric cancer; BMI, body mass index; SD, standard deviation.

^a^ ANOVA test for continuous variables; Chi-square test for categorical variables.

**Table 2:** Baseline characteristics of the HEXA-G study population included in the analysis for GC and BMI at 35 years (Women).

Characteristics	Women (N = 80,361)										
	<18.5 kg/m^2^	(%)	18.5–23.0 kg/m^2^	(%)	23.0–25.0 kg/m^2^	(%)	25.0–29.9 kg/m^2^	(%)	≥30.0 kg/m^2^	(%)	*p* ^a^
Number of participants	4,569	(5.7)	49,915	(62.1)	15,647	(19.5)	9,458	(11.8)	772	(1.0)	<0.001
Number of participants BMI at age 18–20	4,091	(5.8)	44,246	(62.5)	13,589	(19.2)	8,172	(11.6)	656	(0.9)	<0.001
Follow-up year (mean ± SD)	8.6	(2.1)	8.7	(2.1)	8.7	(2.1)	8.6	(2.0)	8.5	(2.1)	0.036
Age (mean ± SD)	49.7	(7.5)	51.3	(7.5)	53.2	(7.6)	53.9	(8.1)	53.5	(8.5)	<0.001
Family history of gastric cancer	372	(8.1)	4,268	(8.6)	1,303	(8.3)	819	(8.7)	73	(9.5)	0.364
Education											<0.001
≤Middle school	984	(21.5)	14,882	(29.8)	6,929	(44.3)	4,901	(51.8)	451	(58.4)	
High school diploma	2,086	(45.7)	23,095	(46.3)	6,369	(40.7)	3,432	(36.3)	257	(33.3)	
≥College degree	1,461	(32.0)	11,505	(23.1)	2,204	(14.1)	1,023	(10.8)	56	(7.3)	
Marital status, Married/cohabitation	3,878	(84.9)	43,547	(87.2)	13,672	(87.4)	8,167	(86.4)	636	(82.4)	<0.001
Smoking status, Ever	282	(6.2)	1,834	(3.7)	493	(3.2)	334	(3.5)	45	(5.8)	<0.001
Alcohol drinking status, Ever	1,547	(33.9)	17,053	(34.2)	5,149	(32.9)	2,854	(30.2)	229	(29.7)	<0.001
Physical activity, Regular exercisers	2,488	(54.5)	24,159	(48.4)	7,438	(47.5)	4,630	(49.0)	410	(53.1)	<0.001
Total energy intake (kcal, mean ± SD)	1698.2	(588.6)	1718.3	(581.3)	1716.2	(575.8)	1709.9	(588.6)	1668.3	(576.9)	0.026

HEXA-G, Health Examinees Study-Gem; GC, gastric cancer; BMI, body mass index; SD, standard deviation.

^a^ ANOVA test for continuous variables; Chi-square test for categorical variables.

Table 3 shows the association between BMI at ages 18–20, 35, and at baseline enrollment period and the risk of GC later in life. Compared to the reference group (BMI 18.5–23.0 kg/m^2^), obesity (BMI ≥30 kg/m^2^) at age 35 was associated with a significantly increased risk of GC (HR 1.94, 95% 1.26–2.97) among the total population. The risk showed a significant linear trend in overweight or obesity groups compared with the normal group (*p* trend 0.01). In a separate analysis by sex, men who were obese (BMI ≥30 kg/m^2^) at age 35 remained significantly associated with increased GC risk (HR 1.79, 95% CI 1.02–3.13, *p* trend 0.05). And in women, obesity (BMI ≥30 kg/m^2^) at age 35 was associated with a significantly increased risk of GC (HR 2.35, 95% CI 1.21–4.60, *p* trend 0.02) as well. Regarding obesity at late adolescence or ages 18–20, no significant association was reported in both men and women. Similarly, regarding the risk of GC according to BMI at baseline, non-significant associations were observed. We further analyzed our data by dividing the BMI category into increments of 2.5 BMI units for participants included in the analysis for BMI at age 35 (S1 Table in S1 File). Here, our findings were consistent with the results found in Table 3.

**Table 3:** Hazard ratios and 95% confidence intervals of gastric cancer according to Asia-Pacific classification of BMI.

Variables		Overall							Men							Women						*p* ^c^
	N	Person-years	GC	%	HR^a^	95% CI		N	Person-years	GC	%	HR^b^	95% CI		N	Person-years	GC	%	HR^b^	95% CI		
**BMI at 18–20 years (kg/m** ^**2**^ **)**																						
	**N = 111,321; GC = 815**							**N = 38,929; GC = 473**							**N = 72,392; GC = 342**							0.33
<18.5	14,466	122431.3	72	0.5	0.96	0.75	1.23	2,123	17959.3	17	0.81	0.76	0.47	1.25	12,343	104472.0	55	0.45	1.03	0.77	1.38	
18.5–23.0	77,210	662517.7	546	0.71	1.00	ref		26,330	225711.7	305	1.17	1.00	ref		50,880	436806.0	241	0.48	1.00	ref		
23.0–25.0	14,841	127407.6	143	0.97	1.04	0.86	1.25	7,791	66978.0	106	1.37	1.04	0.83	1.30	7,050	60429.5	37	0.53	1.01	0.71	1.43	
≥25.0	4,804	41041.6	54	1.13	1.14	0.86	1.51	2,685	22901.6	45	1.69	1.24	0.91	1.70	2,119	18140.0	9	0.43	0.78	0.40	1.52	
*p* trend					0.42							0.10							0.63			
**BMI at 35 years (kg/m** ^**2**^ **)**																						
	**N = 122,724; GC = 927**							**N = 42,363; GC = 531**							**N = 80,361; GC = 396**							0.92
<18.5	5,083	43589.3	18	0.35	0.72	0.45	1.16	514	4451.3	NR^d^	0.78	0.69	0.26	1.86	4,569	39138.0	14	0.31	0.73	0.42	1.25	
18.5–23.0	67,731	586549.1	445	0.66	1.00	ref		17,816	154422.7	214	1.21	1.00	ref		49,915	432126.4	231	0.47	1.00	ref		
23.0–25.0	28,840	248990.3	260	0.9	1.10	0.94	1.28	13,193	113559.7	171	1.31	1.09	0.89	1.33	15,647	135430.6	89	0.58	1.15	0.90	1.47	
25.0–29.9	19,581	168624.8	182	0.93	1.09	0.92	1.30	10,123	86898.2	129	1.29	1.11	0.89	1.38	9,458	81726.6	53	0.57	1.09	0.81	1.48	
≥30.0	1,489	12676.1	22	1.48	1.94	1.26	2.97	717	6084.5	13	1.82	1.79	1.02	3.13	772	6591.7	9	1.18	2.35	1.21	4.60	
*p* trend					0.01							0.05							0.02			
**BMI at baseline survey(kg/m** ^**2**^ **)**																						
	**N = 134,130; C = 1,041**							**N = 45,569; GC = 584**							**N = 88,561; GC = 457**							0.86
<18.5	2,329	19711.5	10	0.43	0.67	0.36	1.26	570	4897.1	5	0.88	0.62	0.26	1.51	1,759	14814.5	5	0.29	0.71	0.29	1.73	
18.5–23.0	51,127	445054.4	346	0.68	1.00	ref		13,018	112986.8	174	1.35	1.00	ref		38,109	332067.5	172	0.46	1.00	ref		
23.0–25.0	37,282	326499.9	290	0.78	0.95	0.81	1.11	13,711	119109.9	154	1.13	0.84	0.68	1.05	23,571	207389.9	136	0.58	1.14	0.91	1.43	
25.0–29.9	39,640	346203.4	368	0.94	1.08	0.93	1.25	17,022	147839.1	239	1.42	1.09	0.90	1.33	22,618	198364.3	129	0.58	1.08	0.85	1.36	
≥30.0	3,752	32323.0	27	0.73	1.00	0.67	1.48	1,248	10725.3	12	0.97	0.88	0.49	1.57	2,504	21597.7	15	0.61	1.20	0.71	2.04	
*p* trend					0.25							0.30							0.30			

BMI, body mass index; N, number of participants; GC gastric cancer; HR, hazard ratio; CI, confidence interval.

^a^ Adjusted for sex, education, smoking status, drinking status, family history of gastric cancer, exercise and total energy intake.

^b^ Adjusted for education, smoking status, drinking status, family history of gastric cancer, exercise and total energy intake.

^c^ *p* interaction for sex difference.

^d^ Frequencies <5 not reported.

## Discussion

In this prospective study of more than 120,000 Koreans, obesity (BMI ≥30 kg/m^2^) in early adulthood, especially at age 35, appears to be significantly associated with elevated risk for GC in later years. Compared with the normal weight (BMI 18.5–23.0 kg/m^2^) group, the association was significant after controlling for epidemiologic risk factors as confounders in both men and women who were obese (*p* interaction for sex difference 0.92).

A few cohort studies have reported an association between excess weight during early adulthood and GC risk. Petrick et al. reported studies on persistent obesity since an early age with the risk of esophageal cancer and GC using cohort data from the U.S. and Denmark [14, 16]. The U.S. study, a pooled analysis of two prospective studies, indicated that prolonged excess weight over the life course, particularly since early adulthood, is associated with increased risks of esophageal and gastric cardia adenocarcinoma (GCA). In line with our research results, this U.S. study highlights the adverse health effects of persistent overweight in early adulthood and supports the potential strategy to reduce GC risk by controlling early adulthood weight. Conversely, a study in Denmark found no association between overweight in early adulthood and GC risk. Although initial results in men with persistent overweight at childhood and early adulthood had shown a 3.2-times increased gastric-esophageal cancer risk, when authors analyzed separately for gastric and esophageal cancer, little evidence was found for increased GC risk in men with overweight during childhood and subsequent early adulthood [16]. Controversial results have been observed in other population cohort studies. Levi et al. found that adolescent obesity was associated with an increased risk for non-cardia GC (HR 1.78; 95%CI 1.12–2.83) in an Israeli population-based cohort study [12]. Similarly, significant associations were found between BMI at 20 years of age and the risk of GCA in a Dutch cohort study; the risk of GCA increased by 7% per 1 kg/m^2^ increase of BMI at age 20 (RR 1.07, 95% CI 1.00–1.15). Additionally, participants with a BMI gain of more than 8 kg/m^2^ had twice the higher risk of GCA with a significantly positive trend [15]. In East Asia, which holds the highest incidence rate for GC, cohort studies have scarcely reported an association between early adulthood BMI and GC risk. A cohort study in Japan has demonstrated an association between high BMI at the age of 20 and increased risk of death from GC [17].

Based on the International Agency for Research on Cancer (IARC) Working Group, there is conclusive evidence that excess body weight is associated with an increased GC risk [28]. However, the mechanisms association between early adulthood BMI and GC remains unclear. One possible explanation is that obesity leads to a higher intra-abdominal pressure, which may result in a higher frequency of gastro-esophageal reflux [29, 30]. Frequent reflux of the gastric acid or bile is closely related to upper gastrointestinal inflammation, ulcer development, intestinal metaplasia and, carcinoma [30, 31]. The exact role of this mechanism yet unclear. One study supported the modest yet statistically significant association between gastro-esophageal reflux and GCA risk [32]. Another possible mechanism is that dysfunctional adipose tissue may lead to metabolic disturbances. Obesity promotes disruptions in multiple metabolic pathways, such as up-regulated sex steroid hormones, insulin, inflammatory mediators and lower adiponectin levels, which influence cell division, cell death and healing [5]. Furthermore, the accumulation of adipose tissue inducing multiple molecular changes could increase cancer cell proliferation and impair apoptosis and result in preneoplastic and neoplastic cell growth [33]. In addition, obesity has been recognized as proinflammatory state leading to elevated level of proinflammatory cytokines, such as TNF and interleukin (IL)-6, which favor the development of cancer [34].

*H*. *pylori* infection has been regarded as an important contributing factor for GC risk [35, 36]. However, we could not take into account its effect in our study due to lack of information. About one-third of adults are infected with *H*. *pylori* worldwide; In the Republic of Korea, its prevalence is estimated to be about 44% in the adult population [37, 38]. Despite its prevalence, epidemiological data lacks support regarding *H*. *pylori* infection and GC in the Republic of Korea. According to a recent case-cohort study from the population-based prospective Korean Multicenter Cancer cohort, a significant U-shaped associations between BMI and GC risk were observed only in the non-infection group [39]. Moreover, a nested case-control study presented no direct association between *H*. *pylori* infection and GC among Koreans [40]. Other studies have reported that certain behavioral and environmental risk factors were associated with GC regardless of *H*. *pylori* infection suggesting that factors such as diet and smoking are not modified by the infection status in relation to gastric carcinogenesis [41, 42]. As obesity is another modifiable factor, we carefully suggest that *H*. *pylori* and early adulthood obesity are not directly correlated with each other in terms of GC development, and, in particular, among Koreans. However, future studies involving *H*. *pylori* infection would be warranted to identify our hypothesis.

The non-significant association between BMI at late adolescence and GC risk may partially be due to different proportions of overweightness and obesity. The prevalence of overweightness, obesity and related diseases is lower in late adolescents than in people in their 30s and above [43, 44]. Likewise, in our supplementary analysis, the proportion of participants with the lowest BMI (<18.5 kg/m^2^) is observed to be greater among late adolescents aged 18–20 years (13.0%, S2 Table in S1 File) compared to young adults aged 35 years (4.1%, Table not shown). In contrast, the proportion of participants with higher BMI category is relatively small among late adolescents that the two categories (overweight 25.0–29.9 kg/m^2^ and obese ≥30.0 kg/m^2^) had to be combined for analytical procedures (S2 Table in S1 File). Some studies have reported that the role of decreased physical activity from adolescence to adulthood during their 30s [45] may play a subtle role in affecting obesity factor for GC risk differences [28, 39]. An additional analyses had been conducted to observe the changes in BMI during young adulthood (between ages 18–20 and 35 years) in its association with GC. Among women only, increased BMI (BMI change >2.0 kg/m^2^ and increase in BMI category change) during this period is significantly associated with GC risk (S3 and S4 Tables in S1 File). Further investigation on changes in weight and obesity-related factors such as BMI using more robust data could help elucidate the relationship between obesity at a young age and GC risk.

There are several limitations in this study. First, we did not perform the GC subgroup analysis according to the anatomic subsites such as gastric non-cardia adenocarcinoma (GNCA) and GCA. Previous studies indicated that GCA showed a greater association with obesity than GNCA [46, 47]. Although we have large cases of GC, there are uncertainties about the anatomic location of GC. Further studies should focus on the BMI at late adolescence and early adulthood with GC risk according to anatomical subsites. Second, as stated before, we were also unable to consider the effect of *H*. *pylori* infection status as we could not obtain this information during the baseline survey. Third, BMI at 18–20 and 35 years of age was calculated using self-reported weight at age 18–20 and 35 years during baseline interview. Therefore, some recall bias may have occurred. In addition, this study was conducted using baseline questionnaire information. As such, we could not consider subsequent changes in diet and several lifestyle factors over time. Some information bias may have consequently occurred in the covariate variables. Finally, even though we adjusted for a wide range of confounders, some residual confounding may remain.

Despite these limitations, our study has several key strengths: A large sample size from one of the largest prospective cohorts in the Republic of Korea; A follow-up window that has begun to accumulate valuable data worthy of investigation; Detailed covariate data with small proportions of missing data; And highly accurate cancer diagnoses from a reliably linked Korean cancer registry data. Additionally, consideration of early age factors, such as BMI at young adulthood in relation to GC risk, minimizes the risk of reverse causality commonly observed in epidemiologic research. Notwithstanding, few case-control studies have reported on this topic in Asia, and these findings allow us to better understand the association between BMI at a younger age and GC among healthy adults in Asia.

In conclusion, our findings provide more evidence for studies on obesity in early adulthood as one of the potential risk factors for GC. Modifiable factors such as obesity in early life may be considered in an effort to understand the etiology of GC.

## Supporting information

S1 File (DOCX)
