# Supplementary material for: Obesity at early adulthood increases risk of gastric cancer from the Health Examinees-Gem (HEXA-G) study
Source: PLoS One. 2022 Feb 4;17(2):e0260826. doi: 10.1371/journal.pone.0260826 (PMC8815964; doi:10.1371/journal.pone.0260826)

| **S1 Table. Hazard ratios and 95% confidence intervals of gastric cancer in increments of 2.5 BMI units among subjects included in the analysis for BMI at 35 years** | | | | | | | | | | | | | | | | | | | | | | | | |
| --- | --- | --- | --- | --- | --- | --- | --- | --- | --- | --- | --- | --- | --- | --- | --- | --- | --- | --- | --- | --- | --- | --- | --- | --- |
| **Variables** |  | **Overall** | | | | | |  |  | **Men** | | | | | |  |  | **Women** | | | | | | ***p*^c^** |
|  | **N** | **Person-year** | **GC** | **%** | **HR^a^** | **95% CI** | |  | **N** | **Person-year** | **GC** | **%** | **HR^b^** | **95% CI** | |  | **N** | **Person-year** | **GC** | **%** | **HR^b^** | **95% CI** | |  |
| **BMI at 35 years (kg/m2)** | | | | | | | |  |  |  |  |  |  |  |  |  |  |  |  |  |  |  |  |  |
|  | **N=122,724; GC=927** | | | | | | |  | **N=42,363; GC=531** | | | | | | |  | **N=80,361; GC=396** | | | | | | | 0.78 |
| ≤18.5 | 5,083 | 43589.3 | 18 | 0.35 | 0.64 | 0.40 | 1.04 |  | 514 | 4451.3 | * | 0.78 | 0.51 | 0.16 | 1.61 |  | 4,569 | 39138.0 | 14 | 0.31 | 0.79 | 0.44 | 1.42 |  |
| 18.6-20.0 | 14,816 | 127826.3 | 82 | 0.55 | 0.91 | 0.71 | 1.17 |  | 2,188 | 19015.6 | 25 | 1.14 | 0.87 | 0.55 | 1.38 |  | 12,628 | 108810.7 | 57 | 0.45 | 1.02 | 0.72 | 1.44 |  |
| 20.1-22.5 | 44,013 | 381617.2 | 283 | 0.64 | 0.72 | 0.72 | 1.00 |  | 12,346 | 107074.3 | 139 | 1.13 | 0.79 | 0.63 | 0.99 |  | 31,667 | 274542.9 | 144 | 0.45 | 0.95 | 0.73 | 1.24 |  |
| 22.6-25.0 | 37,785 | 326465.5 | 340 | 0.90 | 1.00 | ref | |  | 16,490 | 142021.2 | 221 | 1.34 | 1.00 | ref | |  | 21,295 | 184444.4 | 119 | 0.56 | 1.00 | ref | |  |
| 25.1-27.5 | 15,094 | 130078.7 | 135 | 0.89 | 0.93 | 0.76 | 1.14 |  | 7,818 | 67106.6 | 97 | 1.24 | 0.89 | 0.69 | 1.16 |  | 7,276 | 62972.2 | 38 | 0.52 | 0.96 | 0.65 | 1.41 |  |
| 27.6-30.0 | 4,444 | 38176.4 | 47 | 1.06 | 1.14 | 0.84 | 1.16 |  | 2,290 | 19662.9 | 32 | 1.4 | 1.10 | 0.73 | 1.64 |  | 2,154 | 18513.5 | 15 | 0.7 | 1.36 | 0.77 | 2.38 |  |
| >30.0 | 1,489 | 12676.1 | 22 | 1.48 | 1.73 | 1.12 | 2.66 |  | 717 | 6084.5 | 13 | 1.8 | 1.82 | 1.04 | 3.20 |  | 772 | 6591.7 | 9 | 1.17 | 1.92 | 0.89 | 4.14 |  |
| *p* trend |  |  |  |  | <0.01 | | |  |  |  |  |  | 0.02 | | |  |  |  |  |  | 0.02 | | |  |
| BMI, body mass index; N, number of participants; GC gastric cancer; HR, hazard ratio; CI, confidence interval | | | | | | | | | | | | | | | | | | | |  |  |  |  |  |
| ^a^ Adjusted for sex, education, smoking status, drinking status, family history of gastric cancer, exercise and total energy intake | | | | | | | | | | | | | | | | | | | |  |  |  |  |  |
| ^b^ Adjusted for education, smoking status, drinking status, family history of gastric cancer, exercise and total energy intake | | | | | | | | | | | | | | | | | | | |  |  |  |  |  |
| ^c^ *p* interaction for sex difference | | |  |  |  |  |  |  |  |  |  |  |  |  |  |  |  |  |  |  |  |  |  |  |
| ^*^ Frequencies <5 not shown | | |  |  |  |  |  |  |  |  |  |  |  |  |  |  |  |  |  |  |  |  |  |  |

| **S2 Table. Baseline characteristics of the HEXA-G study population included in the analysis for BMI at 18-20 years** | | | | | | | | | |
| --- | --- | --- | --- | --- | --- | --- | --- | --- | --- |
| **Characteristics** | **Overall (N=111,321)** | | | | | | | | |
|  | <18.5 | (%) | 18.5-23.0 | (%) | 23.0-25.0 | (%) | ≥25.0 | (%) | *p* |
| Number of participants | 14,466 | 13.0 | 77,210 | 69.4 | 14,841 | 13.3 | 4,804 | 4.3 | <0.001 |
| Number of participants with BMI at age 35 | 14,172 | 13.0 | 75,630 | 69.4 | 14,508 | 13.3 | 4,686 | 4.3 | <0.001 |
| Follow-up year (mean ± SD) | 8.5 | 2.1 | 8.6 | 2.1 | 8.6 | 2.1 | 8.5 | 2.0 | <0.001 |
| Age (mean ± SD) | 50.0 | 7.4 | 51.9 | 7.8 | 54.1 | 8.1 | 55.3 | 8.3 | <0.001 |
| Gender, Men | 2,123 | 14.7 | 26,330 | 34.1 | 7,791 | 52.5 | 2,685 | 55.9 | <0.001 |
| Family history of gastric cancer | 1,170 | 8.1 | 6,509 | 8.4 | 1,282 | 8.6 | 396 | 8.2 | 0.036 |
| Education |  |  |  |  |  |  |  |  | <0.001 |
| ≤Middle school | 2,857 | 19.8 | 20,814 | 27.0 | 5,303 | 35.7 | 2,103 | 43.8 |  |
| High school diploma | 6,852 | 47.4 | 34,701 | 44.9 | 5,856 | 39.5 | 1,659 | 34.5 |  |
| ≥College degree | 4,670 | 32.3 | 21,102 | 27.3 | 3,569 | 24.1 | 1,002 | 20.9 |  |
| Marital status, Married/cohabitation | 12,791 | 88.4 | 69,476 | 90.0 | 13,449 | 90.6 | 4,237 | 88.2 | <0.001 |
| Smoking status, Ever | 2,109 | 14.6 | 21,067 | 27.3 | 5,972 | 40.2 | 2,029 | 42.2 | <0.001 |
| Alcohol drinking status, Ever | 6,116 | 42.3 | 38,427 | 49.8 | 8,582 | 57.8 | 2,749 | 57.2 | <0.001 |
| Physical activity, Regular exercisers | 7,191 | 49.7 | 35,442 | 45.9 | 6,676 | 45.0 | 2,292 | 47.7 | <0.001 |
| Total energy intake (kcal, mean ± SD) | 1770.8 | 575.2 | 1757.4 | 587.0 | 1793.7 | 569.6 | 1814.6 | 623.9 | <0.001 |
| HEXA-G, Health Examinees Study-Gem; BMI, body mass index; SD, standard deviation | | | | | | | | | |
| ^a^ ANOVA test for continuous variables; Chi-square test for categorical variables | | | | | | | | | |

| **S3 Table. Hazard ratios and 95% confidence intervals of gastric cancer according to BMI change (kg/m^2^) during young adulthood (between ages 18-20 and 35 years)** | | | | | | | | | | | | | | | | | | | | | |
| --- | --- | --- | --- | --- | --- | --- | --- | --- | --- | --- | --- | --- | --- | --- | --- | --- | --- | --- | --- | --- | --- |
| **BMI change** | **Overall** | | | | | |  | **Men** | | | | | |  | **Women** | | | | | |  |
|  | **GC** | | | **HR^a^** | **95% CI** | |  | **GC** | | | **HR^b^** | **95% CI** | |  | **GC** | | | **HR^c^** | **95% CI** | | ***p^d^*** |
|  | **No** | **Yes** | **(%)** |  |  |  |  | **No** | **Yes** | **(%)** |  |  |  |  | **No** | **Yes** | **(%)** |  |  |  |  |
|  | **N=107,959; GC=794** | | |  |  |  |  | **N=37,938; GC=462** | | |  |  |  |  | **N=70,021; GC=332** | | |  |  |  | 0.25 |
| Minimal  (>-1.0 to ≤2.0 kg/m^2^) | 59,512 | 451 | (56.8) | 1.00 | ref |  |  | 21,791 | 283 | (61.3) | 1.00 | ref |  |  | 37,721 | 168 | (50.6) | 1.00 | ref |  |  |
| Decreased  (≤-1.0 kg/m^2^) | 8,669 | 55 | (6.9) | 1.05 | 0.80 | 1.40 |  | 1,800 | 30 | (6.5) | 1.16 | 0.80 | 1.69 |  | 6,869 | 25 | (7.5) | 0.94 | 0.62 | 1.43 |  |
| Increased  (>2.0 kg/m^2^) | 38,984 | 288 | (36.3) | 1.17 | 1.01 | 1.36 |  | 13,885 | 149 | (32.3) | 1.11 | 0.91 | 1.35 |  | 25,099 | 139 | (41.9) | **1.30** | **1.04** | **1.63** |  |
| *p* trend |  |  |  | 0.09 | | |  |  |  |  | 0.59 | | |  |  |  |  | **0.02** | | |  |
| BMI, body mass index; GC gastric cancer; HR, hazard ratio; CI, confidence interval | | | | | | | | | | | | | | | | | | | | | |
| ^a^ Adjusted for sex, education, smoking status, drinking status, family history of gastric cancer, exercise and total energy intake | | | | | | | | | | | | | | | | | | | | | |
| ^b^ Adjusted for education, smoking status, drinking status, family history of gastric cancer, exercise and total energy intake | | | | | | | | | | | | | | | | | | | | | |
| ^c^ Adjusted for education, drinking status, family history of gastric cancer, exercise and total energy intake | | | | | | | | | | | | | | | | | | | | | |
| ^d^ *p*-interaction for sex difference | | | | | | | | | | | | | | | | | | | | | |

**S4 Table. Hazard rations and 95% confidence intervals of gastric cancer according to changes in BMI category**
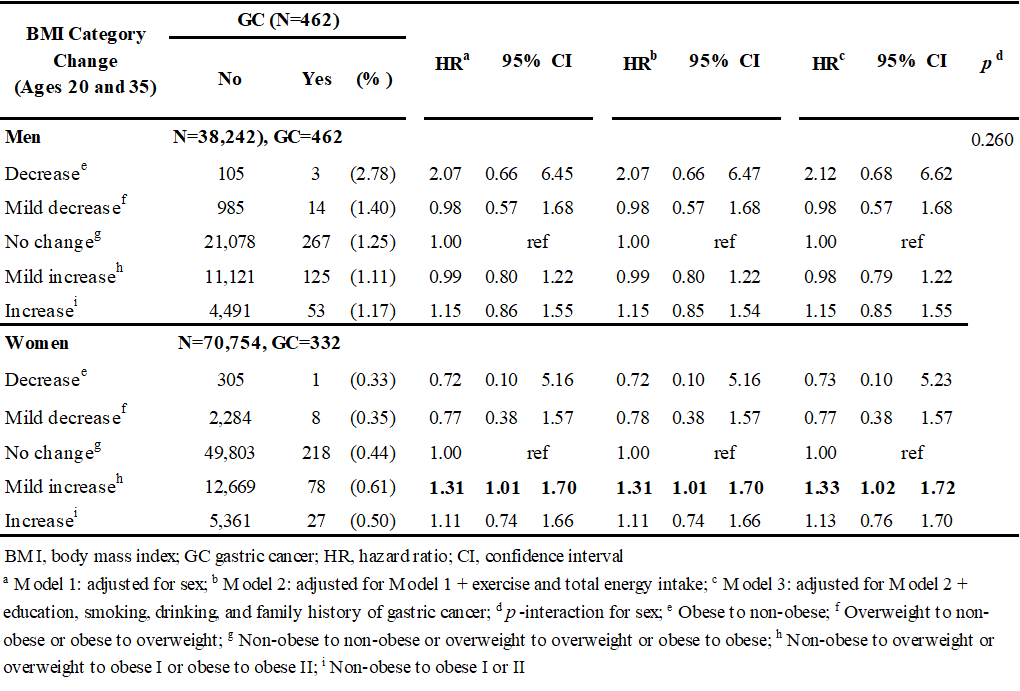

Supplement: S1 File — (DOCX) [file pone.0260826.s001.docx]
